# Analysis of Complex Patterns of Human Exposure and Immunity to *Schistosomiasis mansoni*: The Influence of Age, Sex, Ethnicity and IgE

**DOI:** 10.1371/journal.pntd.0000820

**Published:** 2010-09-14

**Authors:** Angela Pinot de Moira, Anthony J. C. Fulford, Narcis B. Kabatereine, John H. Ouma, Mark Booth, David W. Dunne

**Affiliations:** 1 Department of Pathology, University of Cambridge, Cambridge, United Kingdom; 2 MRC International Nutrition Group, London School of Hygiene and Tropical Medicine, London, United Kingdom; 3 Vector Control Division, Ministry of Health, Kampala, Uganda; 4 Kenya Methodist University, Meru, Kenya; 5 Wolfson Research Institute, Durham University Queen's Campus, Stockton on Tees, United Kingdom; Leeds University, United Kingdom

## Abstract

**Background:**

Numerous factors may influence *Schistosoma* infection intensity and prevalence within endemic communities, including exposure-related factors such as local environment and behaviour, and factors relating to susceptibility to infection such as immunology and genetics. While animal studies performed in the laboratory can be tightly controlled, human populations are highly heterogeneous, varying according to demographic characteristics, genetic background and exposure to infection. The heterogeneous nature of human water contact behaviour in particular makes it difficult to distinguish between a lack of cercarial exposure and reduced susceptibility to infection as the cause for low levels of infection in the field.

**Methods and Principal Findings:**

In this study we investigate risk factors for *Schistosoma mansoni* infection in a rural Ugandan fishing community receiving treatment as part of a multi-disciplinary longitudinal reinfection study. More specifically, we examine the influence that age, sex and ethnic background have on susceptibility to reinfection after anti-helminth drug treatment, but use individual estimates of cercarial exposure and multivariable methods in an attempt to remove noise created by environmental and behavioural heterogeneities. We then investigate whether schistosome-specific IgE immune responses could account for any remaining variations in susceptibility to reinfection. Our findings suggest that observed ethnic- and sex-related variations in *S. mansoni* reinfection were due to variations in cercarial exposure, as opposed to biological differences in susceptibility to infection. Age-related differences in reinfection were not explained by exposure, however, and appeared linked to the balance of IgE and IgG_4_ to the tegumental antigen SmTAL1 (formerly Sm22.6), which itself was significantly related to resistance to reinfection.

**Conclusions:**

This study highlights the benefit of taking a multidisciplinary approach in complex field settings; it allows the ecology of a population to be understood and thus more robust conclusions to be made.

## Author Summary

Human schistosomiasis is a chronic parasitic disease affecting around 200 million people worldwide. Infection occurs when cercariae penetrate the skin during water contact. Although there is effective treatment for schistosomiasis, individuals remain susceptible to reinfection after treatment; some individuals, however, appear more susceptible to reinfection than others. The highly heterogeneous nature of human water contact behaviour makes it difficult to identify whether low levels of reinfection are caused by immunity or a simple lack of cercarial exposure; this complicates the characterisation of risk factors for infection and immune correlates of protection. Here, we take a multidisciplinary approach using individual estimates of cercarial exposure and multivariable analysis to allow for environmental and behavioural heterogeneities. We examine the influence of demographic factors and antibody responses on susceptibility to reinfection. While observed ethnic- and sex-related variations in *Schistosoma mansoni* reinfection could be explained by differences in exposure, age-related differences appeared linked to the balance of specific IgE and IgG4 antibodies, themselves related to resistance to reinfection. Our study highlights the benefits of a multidisciplinary approach in complex field settings: it improves our understanding of a population's ecology and therefore the biology of disease.

## Introduction

Despite numerous control efforts, the estimated worldwide prevalence of schistosomiasis has not changed over the past 50 years [1], [2]. More than 200 million people are currently thought to be infected with *Schistosoma* spp. [3], with the majority of these infections occurring amongst the world's poorest populations in sub-Saharan Africa. Although in more recent years the establishment of a number of national control programmes offering chemotherapeutic treatment with praziquantel has helped to reduce the burden of schistosomiasis [4], [5], it is very difficult to halt transmission solely through drug treatment [6]. This is because, like with many other human helminth infections, individuals remain susceptible to reinfection after treatment. Patterns of reinfection in communities that have received curative chemotherapy are, however, never uniform, with some individuals appearing more susceptible to reinfection and others appearing comparably resistant to reinfection.

Unfortunately, the heterogeneous nature of human water contact behaviour, and therefore cercarial exposure (the infectious stage of the parasite), impedes our ability to distinguish between immunity and simply a lack of cercarial exposure as the root cause for low levels of infection. For example, the characteristic age-infection curves observed in schistosomiasis-endemic communities, whereby infection intensities peak in early adolescence and decline thereafter, is believed to be evidence that acquired immunity to infection can develop. In support of this, IgE antibody levels to worm antigens, which have been linked to resistance to reinfection [7]–[13], tend to increase with age, whereas antibody levels to egg antigens generally decline or are unchanged [14]–[16]. However, an alternative explanation is that, rather than being immune-mediated, age-related changes in infection and infection intensity are driven by exposure: studies investigating water contact behaviour in endemic communities have observed that water contacts generally decline with age [17]. Although post-adolescent declines in infection intensities have been observed in populations where adults remain heavily exposed after childhood [18], this was in an ecological study where the unit of observation was the population rather than the individual.

Numerous studies have also documented differences in schistosomiasis infection prevalence or intensity between males and females living in the same community, with males frequently having heavier infections than females (for example, [19]–[24]). Gender-related differences in infection prevalence or intensity are frequently attributed to socio-cultural or behavioural factors [20], [23], [24], but there is evidence that they may also be related to differences in susceptibility to infection. Studies in CBA/J mice have demonstrated increased susceptibility to infection amongst female mice [25], which appears related to testosterone levels during early infection [26]. Furthermore, in human studies, gender-related differences in the magnitude of the humoral immune responses have been reported for the three most prevalent species of *Schistosoma* that infect man [14], [27].

Although historically less debated, ethnic differences in infection have also been described in a number of communities, and as with observed sex differences, have been mainly ascribed to differences in water contact behaviour, relating to occupational or cultural differences [28]–[31]. Unfortunately, in the majority of these studies, inferences were drawn mainly from anecdotal water contact evidence and without systematic water contact observations. In one study conducted in a Ugandan fishing community, Kabatereine and colleagues ruled out greater exposure to infection as an explanation for the heavier infection intensities observed amongst the Alur people, and instead speculated that their heavier infections were due to genetic differences which increased their susceptibility to infection[20]. This suggestion is supported by other epidemiological studies investigating patterns of exposure and reinfection in schistosomiasis-endemic communities which have identified individuals that appear to be more resistant or pre-disposed to infection [32]–[34]. In addition, several human genetic studies have identified genes that are associated with relative susceptibility or immunity to infection (reviewed in Campino *et al.* [35]).

Reinfection studies involving the chemotherapeutic treatment of infected individuals and the monitoring of subsequent reinfection have identified a number of immune responses potentially important for resistance to human schistosomiasis. High levels of IgE against adult worm antigens have been associated with resistance to reinfection [8], [10], [11], [36], [37], whereas high levels of IgG_4_ against adult worm and egg antigens have been associated with susceptibility to reinfection [10], [11], [13], [38]. Evidence suggests that it may be the balance of IgE to IgG_4_ that determines resistance/susceptibility to reinfection in humans [10], [13], [39] and that IgG_4_ may attenuate the protective effect of IgE [38]. IgE directed at particular antigens appears to correlate more strongly with subsequent lower reinfection intensities, for example, IgE responses directed at adult worm tegumental antigens and in particular the 22.6 kDa antigen Tegument-Allergen-Like1 (SmTAL1, formerly Sm22.6; NCBI Acc. No. AAA29922), have been associated with resistance to reinfection in schistosomiasis mansoni*-*endemic communities [8], [12]. Sequence homologues to SmTAL1 have been identified in *S. japonicum* [14], [40] and *S. haematobium* [41], and both are targets for human IgE responses.

One approach to understanding determinants of susceptibility to infection is to construct estimates of cercarial exposure that capture variations in behaviour and environmental influences, and adjust for these using multivariable methods. In the current study we use data from a multidisciplinary reinfection study based in a Ugandan fishing community highly endemic for schistosomiasis [42] to investigate variations in *S. mansoni* reinfection. To allow for the highly complex, strongly ethnically-influenced patterns of exposure observed in this community [43], we utilise previously derived individual-level cercarial exposure scores [43] to adjust for variations in exposure. These exposure scores incorporate both behavioural and environmental elements of exposure by combining data from systematic water contact observations (duration of contact and degree of immersion) with malacological data (site-specific snail infection intensities and species-specific diurnal shedding patterns). Using these exposure scores, we first investigate the roles of age and sex in susceptibility to *Schistosoma* infection and, as Kabatereine and colleagues have speculated [20], whether the Alur ethnic group are more susceptible to infection. We then explore whether schistosome-specific IgE or IgG_4_ immune responses could account for any remaining variation in susceptibility to reinfection. By taking a multidisciplinary approach to more precisely capture and adjust for variations in exposure, we demonstrate the importance of considering exposure patterns whilst undertaking population-based studies on risk factors for infection and the biology of disease. This multidisciplinary approach can lead to more robust conclusions, even in complex settings such as ours, where the confounding issue of exposure would otherwise be a major limitation.

## Methods

### Study area and population

The current study was conducted in the fishing village of Booma, located in the parish of Butiaba, Masindi district, north-western Uganda. To our knowledge the community had not received treatment for schistosomiasis before the study. Full details of study area and population have been described elsewhere [20], [43], briefly, the village is located along the eastern shore of Lake Albert where conditions are unsuitable for agriculture and the main source of income is through the fishing industry. The lake offers the main source of water in the village, providing water for both domestic and personal needs. There are two predominant ethno-linguistic backgrounds in Booma; Bantu-linguistic Bagungu and Nilotic-linguistic Alur. Whilst the Bagungu are indigenous to the region, the Alur are immigrant to the area and originate either from the West Nile region in Uganda, or from the Democratic Republic of the Congo. The two ethno-linguistic groups live at geographically distinct ends of the village (approximately 1 km apart) and there are few intertribal marriages.

### Parasitological surveys

A demographic survey of the study village was conducted and a register drawn up of all inhabitants by household. From this a random sample of 290 individuals aged between 5 and 66 years and either born in the village or resident for 10 or more years was selected. The current investigation focuses on the Alur and Bagungu ethnic groups, who made up 96% of the study sample (278 individuals in total). Starting in July 1998, study members were treated twice, two weeks apart, with 40 mg/kg body weight of praziquantel. Parasitological surveys were conducted in May 1998, prior to treatment, and 5 weeks, 12 months, 18 months and 22 months after the second treatment. Since water contact was only followed for 11 months following treatment, we limited our analysis to reinfection at 12 months. 5 week stool samples were used to detect treatment failure or non-compliance. Pre- and post- treatment stool samples were collected on three consecutive days, and for each stool sample, two 50 mg Kato-Katz [44] slides were prepared and examined microscopically.

### Cercarial exposure scores

Systematic water contact observations conducted on the whole community between mid November 1998 and early September 1999 at 19 different sites were used to estimate study participants' water contact duration. Full details of these observations are given elsewhere [43]. Since duration of water contact is not an accurate measure of cercarial exposure, individual water contact durations were weighted so as to reflect the risk associated with the time of day, site of contact and the degree of immersion. Overall exposure scores were thus assigned to each individual using the following function [45]:
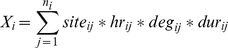



Where *X_i_* is the exposure score for the *i^th^* individual, observed *n_i_* times, with the *j^th^* contact lasting *dur_ji_* minutes. *Site_ji_*, *hr_ji_* and *deg_ji_* are the respective site, time of day and degree of immersion weightings for the *j^th^* contact. Full details of these weightings are given in [43]; briefly, malacological data collected in conjunction with water contact data (Kabatereine, unpublished data) were used to generate weightings for each site (*site_ji_*), which described the snail infection intensities and the relative abundance of *Biomphalaria stanleyi* vs. *B. sudanica* at each site, the former of which appears to be a more susceptible host and have a greater capacity as an intermediate host [46]. Time of day weightings (*hr_ji_*) reflected the diurnal rhythms of cercarial shedding and were constructed using a bell-curve function [47] fitted using cercarial shedding data collected as part of a detailed malacological study [48]. Degree of immersion weightings used a simple weighting system developed by Fulford and colleagues which counts the number of body parts immersed [49].

Spearman-Brown split-half coefficients [reviewed by Fulford [45], Chapter 3] were calculated in order to assess the reliability of water contact observations and the derived exposure scores. Reliability estimates were high: Spearman-Brown split half reliability coefficients were 0.96, 0.97 and 0.93 for total duration, frequency of contact and total exposure, respectively.

### IgE and IgG_4_ levels

Archived data from isotype-specific ELISAs from the Booma study [50] were used to analyse associations between IgE and IgG_4_ levels to *S. mansoni* soluble adult worm antigen (SWA), adult worm tegumental antigen (Teg), the adult worm tegumental antigen SmTegument-Allergen-Like-1 (SmTAL1, formerly Sm22.6; NCBI Acc. No. AAA29922) and soluble egg antigen (SEA). These assays used sera prepared from venous blood samples donated 7 weeks after first treatment (5 weeks after second treatment). Antigens were prepared as previously described [8], [12] and post-treatment human sera samples were assayed at optimal concentrations of 1 in 200 (IgG_4_) and 1 in 20 (IgE). Sera samples were randomly distributed on 96 well microtiter plates, in triplicate and on separate plates. Samples from healthy European controls were also assayed and acted as a standard. Triplicate assays were averaged using an in-house merger programme, which adjusts for plate-to-plate variation by comparing ODs to the standard control serum.

### Statistical analysis

All analyses were restricted to individuals from the Alur or Bagungu ethnic groups. As explained previously, because water contact behaviour was only followed for 11 months following treatment, we restricted analyses to reinfection 12 months after treatment so as not to bias results. Due to the low levels of reinfection observed in our study sample at 12 months (only half of individuals were reinfected at this time point), there was insufficient power to model intensities of reinfection; analyses were therefore restricted to a binary outcome (reinfected vs. not reinfected) and modelled using logistic regression. The possibility of using zero-inflated negative binomial models to model our data was explored; only the logit coefficients for predicting excess zeros were significant in these models.

An initial descriptive analysis explored patterns of infection and reinfection at baseline for each of our demographic variables (ethnicity, sex and age). Infection with *S. mansoni* was defined as having at least one detectable *S. mansoni* egg in Kato-Katz smears. Intensity of infection was determined by multiplying the number of eggs per slide by a factor of 20 to estimate eggs per gram of faeces (epg), and then taking the arithmetic mean epg for each individual. Since egg counts were positively skewed, geometric means (GM) were calculated to explore differences in infection intensities, with 1 added to all egg counts to remove zero values (epg+1).

For post-treatment data, we first conducted an initial univariable analysis exploring associations between our independent variables (ethnicity, sex, age, duration of water contact, estimated cercarial exposure and levels of IgE and IgG_4_ to SWA, tegument, SmTAL1 and SEA) and reinfection using logistic regression. Multivariable logistic regression models were then constructed to examine risk factors further. These models were initially fitted without duration of water contact, level of cercarial exposure or post-treatment antibody levels fitted, which were fitted in subsequent models. IgE and IgG_4_ isotypes were included together in multivariable logistic models, so as to explore associations between the balance of IgE vs. IgG_4_ and *S. mansoni* reinfection. After fitting each multivariable model, all possible interactions were tested and, if found to be significant (*P*<0.05), were also included in models. Both univariable and multivariable logistic regression models excluded individuals with detectable *S. mansoni* eggs in Kato-Katz smears at 5 weeks; excluding these individuals did not alter the final conclusions (Supporting Tables S1).

For all analyses, likelihood ratio tests were used to determine statistical significance. Age was classified into seven groups: 7–9 years, 10–12 years, 13–16 years, 17–23 years, 24–30 years, 31–38 years and 39–50 years. Duration of water contact, cercarial exposure scores and OD values from ELISAs were all analysed as the logarithm, with 1, 0.01 and 0.03 added respectively to remove zero values. All analyses were conducted using Stata 10.1 (Stata Corporation, Texas, USA).

### Ethics statement

Ethical approval for the study was obtained from the Uganda National Council for Science and Technology (UNCST) and cleared by the Office of the President. The study was also supported by the Cambridge Local Research Ethics Committee. Prior to enrolment in the study, the study was explained to each adult or parent/guardian of each child selected for the study and verbal consent obtained. Verbal informed consent was sought because of the high level of illiteracy in Booma and because the predominant language, Lougungu, is not a written language. This method was approved by the ethical review committee of the UNCST. In order to document verbal consent, the name of each individual providing consent was recorded.

## Results

### Study compliance

A total of 290 individuals were selected at random for the Booma reinfection study and of these 278 (96%) were either Alur or Bagungu. Thirteen individuals (of the 278, 5%) refused to participate in the study, a further two individuals failed to comply with treatment (indicated by an increased egg count at 5 weeks following treatment), and one individual had an extreme outlying duration of water contact; these individuals were excluded from further analysis. Of the remaining 262 study members, 115 (43.9%) were Alur and 147 (56.1%) were Bagungu. Treatment uptake rates and the average number of stool samples provided by study members at baseline and follow-up surveys were compared between ethnic groups: there were no notable differences between the Alur and Bagungu ethnic groups. Study participants provided an average number of 2.99 stools at baseline and 2.91 stools at 12 months.

### *S. mansoni* infection at baseline

**Figure 1:**
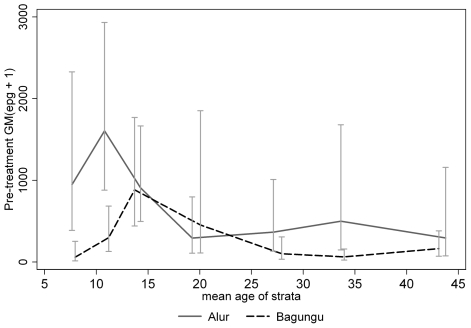
Geometric mean egg counts (epg+1) over age by ethnic group before treatment (baseline).

Prevalence and intensity of infection was high in Booma before treatment: 93.4% (CI_95%_: 89.7%, 96.1%) of study participants were infected and overall GM infection intensity (epg+1) was 267.09 (CI_95%_: 200.48, 355.82). Infection varied by ethnic group, with both prevalence and intensity of infection being greater in the Alur than in the Bagungu: 98.2% (CI_95%_: 93.6%, 99.8%) of Alur vs. 89.8% (CI_95%_: 83.7%, 94.2%) of Bagungu were infected with *S. mansoni*, and GM infection intensity was 564.25 (CI_95%_: 395.92, 804.14) in the Alur compared with 151.84 (CI_95%_: 101.13, 227.99) in the Bagungu. Infection intensity, but not prevalence, also varied significantly with age. GM egg counts followed a convex relationship with age: GM infection intensity peaked in early adolescence and was notably lower amongst adults (Figure 1). However, whereas the peak in GM infection intensity occurred at around the age of 10 years in the Alur, GM egg count peaked at around 14 years in the Bagungu (Figure 1).

### *S. mansoni* infection at 12 months post treatment

**Figure 2:**
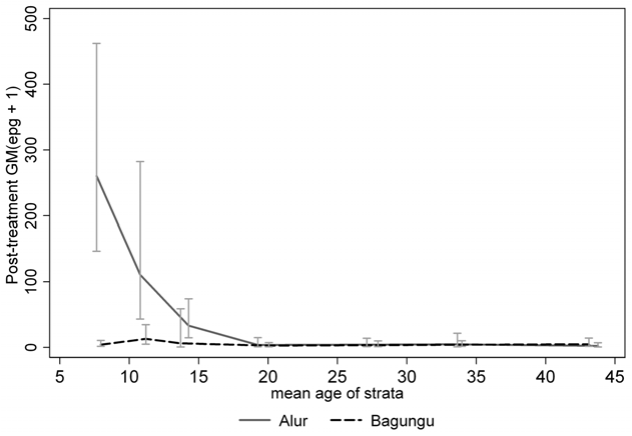
Geometric mean egg counts (epg+1) over age by ethnic group 12 months after treatment.

Treatment resulted in substantial reductions in prevalence and GM egg counts. The overall cure rate in Booma was 81.1% and the GM egg count reduction was 95%. Reinfection after treatment was relatively slow: only 50.9% (CI_95%_: 44.0%, 57.8%) of study participants were reinfected 12 months after treatment, at which point GM infection intensity was only 7.67 (CI_95%_: 5.63, 10.45). Non-immune-related factors associated with reinfection are given in Table 1. Reinfection was significantly more likely in males [OR = 2.17 (CI_95%_: 1.14, 4.13)], but less likely in the Bagungu [OR = 0.54 (0.28, 1.04)] and also varied with age, peaking in the 10–12 year olds and declining with age thereafter. As for before treatment, reinfection peaked earlier in the Alur than the Bagungu (≈8 years vs. ≈11 years; Figure 2). Duration of water contact and cercarial exposure score were also strongly associated with reinfection (*P*<0.0001; Table 1).

**Table 1:** Associations between potential risk factors and *S. mansoni* reinfection 12 months after treatment†.

		N (%) or GM (GSD)*	N (%) reinfected	OR** (CI_95%_)	*P*-value
**Ethnic group**	Alur	57 (35.0)	29 (50.9)	1.00	
	Bagungu	106 (65.0)	38 (35.9)	0.54 (0.28, 1.04)	0.06
**Sex**	Female	74 (45.4)	23 (31.1)	1.00	
	Male	89 (54.6)	44 (49.4)	2.17 (1.14, 4.13)	0.02
**Age (years)**	7–9	15 (9.2)	7 (46.7)	1.00	
	10–12	15 (9.2)	9 (60.0)	1.71 (0.40, 7.29)	
	13–16	16 (9.8)	11 (68.8)	2.51 (0.58, 10.88)	
	17–23	23 (14.1)	10 (43.5)	0.88 (0.24, 3.25)	
	24–30	30 (18.4)	10 (33.3)	0.57 (0.16, 2.03)	
	31–38	35 (21.5)	14 (40.0)	0.76 (0.23, 2.58)	
	39–50	29 (17.8)	6 (20.7)	0.30 (0.08, 1.16)	
**Water contact duration^¶a^**		84.34 (6.4)		4.19 (2.50, 7.04)	<0.0001
**Cercarial exposure** ‡ **^a^**		1.08 (25.9)		4.80 (2.89, 7.95)	<0.0001

**†:** Individuals with detectable eggs at 5 weeks excluded.

*GM  =  geometric mean, GSD  =  geometric standard deviation.

**OR  =  odds ratio.

**¶:** Ln (minutes +1).

a ORs are per unit S.D. increase.

**‡:** Ln(exposure units +0.01); derived by weighting water contact duration by the average number of infected snails at water contact site, time of day and degree of immersion.

Associations between post-treatment IgE and IgG_4_ antibody responses to adult worm and egg antigens, and reinfection are given in Table 2. Although no significant associations were found, whereas higher levels of IgE to SmTAL1 were associated with reduced odds of reinfection, stronger IgG_4_ responses to all four antigens were associated with increased odds of reinfection.

**Table 2:** Associations between post-treatment IgE and IgG4 against worm and egg, and 12 month reinfection†.

		OR* (CI_95%_)	*P*-value
**IgE** **			
	SWA	1.14 (0.73, 1.77)	0.57
	Tegument	1.16 (0.71, 1.91)	0.55
	SmTAL1	0.96 (0.69, 1.33)	0.81
	SEA	1.75 (0.71, 4.36)	0.23
**IgG4** **			
	SWA	1.17 (0.78, 1.76)	0.45
	Tegument	1.11 (0.64, 1.94)	0.71
	SmTAL1	1.10 (0.80, 1.51)	0.55
	SEA	1.07 (0.54, 2.11)	0.85

**†:** Individuals with detectable eggs at 5 weeks excluded.

*OR  =  odds ratio.

**OR per unit increase; antibody levels were estimated by isotype-specific ELISA, OD values were log-transformed with 0.03 added to remove zeros.

### Risk factor analysis

To assess whether the observed demographic variations in reinfection could be due to differences in susceptibility to infection, further analyses used multivariable models to investigate whether differences in reinfection could be explained by differences in water contact behaviour or cercarial exposure (Table 3). In an attempt to try to understand the relative roles of water contact behaviour and cercarial exposure in reinfection more fully, duration of water contact and level of cercarial exposure were initially excluded from these models. Likelihood of reinfection was significantly lower amongst the Bagungu compared with the Alur [OR = 0.08 (CI_95%_: 0.02, 0.27), *P*<0.0001] and borderline significantly lower amongst males compared with females [OR = 0.32 (CI_95%_: 0.10, 1.03), *P* = 0.05]; however, significant interactions were found between ethnic group and sex (*P*<0.0001), suggesting that associations between sex and reinfection differed in the two ethnic groups. Reinfection was borderline significantly associated with age (*P* = 0.05, Table 3), but there was evidence that this association also varied by ethnic group (ethnic group-age interaction term significant, *P* = 0.003, results not shown).

**Table 3:** Results from multivariable logistic models examining associations between demographic factors and 12 month reinfection*.

	Model 1^a^		Model 2^b^		Model 3^c^	
	OR** (CI_95%_)	*P*-value	OR** (CI_95%_)	*P*-value	OR** (CI_95%_)	*P*-value
**Ethnic group (Bagungu)**	0.08 (0.02, 0.27)	<0.0001	0.14 (0.04, 0.53)	0.002	0.31 (0.08, 1.23)	0.09
**Sex (Male)**	0.32 (0.10, 1.03)	0.05	0.66 (0.18, 2.46)	0.54	0.39 (0.11, 1.39)	0.14
**Ethnicity x sex interaction**	27.18 (5.67, 130.35)	<0.0001	6.80 (1.13, 40.88)	0.04	4.65 (0.75, 28.83)	0.10
**Age** d						
7–9 years	1.00		1.00		1.00	
10–12 years	1.17 (0.23, 6.08)		1.40 (0.23, 8.49)		0.51 (0.06, 4.37)	
13–16 years	1.85 (0.34, 10.02)		1.63 (0.27, 9.98)		0.67 (0.08, 5.35)	
17–23 years	0.72 (0.16, 3.26)		0.95 (0.18, 5.03)		0.37 (0.06, 2.42)	
24–30 years	0.38 (0.09, 1.64)		0.32 (0.06, 1.62)		0.13 (0.02, 0.85)	
31–38 years	0.43 (0.10, 1.73)		0.31 (0.06, 1.50)		0.15 (0.02, 0.93)	
39–50 years	0.22 (0.05, 1.03)	0.05	0.19 (0.03, 1.03)	0.03	0.07 (0.01, 0.51)	0.02
**Water contact duration** † **^e^**			3.87 (2.18, 6.90)	<0.0001		
**Cercarial exposure** ‡ **^e^**					5.12 (2.71, 9.66)	<0.0001

*Models examined associations between ethnicity, sex and age, and *S. mansoni* reinfection 12 months after treatment. Model 1 is without adjustment for behaviour or cercarial exposure, Model 2 adjusts for observed water contact duration and Model 3 adjusts for estimated cercarial exposure.

a Model included ethnic group, sex and age; excludes individuals with detectable eggs at 5 weeks.

b Model included ethnic group, sex, age and observed water contact duration; excludes individuals with detectable eggs at 5 weeks.

c Model included ethnic group, sex, age and cercarial exposure score; excludes individuals with detectable eggs at 5 weeks.

**OR  =  odds ratio.

d 6 degrees of freedom used to fit age.

**†:** Ln (minutes +1).

e OR per unit S.D. increase.

**‡:** Ln(exposure units +0.01); derived by weighting water contact duration by water contact site, time of day and degree of immersion.

This ethnic group-age group interaction was lost once duration of water contact was allowed for in analysis (*P* = 0.07), suggesting that any differences in the effect of age on reinfection observed in the two ethnic groups were due to differences in duration of water contact. After adjusting for duration of water contact (Table 3), sex was no longer a significant risk factor for reinfection [OR = 0.66 (CI_95%_: 0.18, 2.46) *P* = 0.54, males vs. females], but ethnicity remained significantly associated with reinfection [OR = 0.14 (CI_95%_: 0.04, 0.53) *P* = 0.002, Bagungu vs. Alur] and the different influence of sex observed in the Alur and Bagungu ethnic groups also remained significant (ethnic group-sex interaction term significant, *P* = 0.04).

When estimated cercarial exposure was included in the logistic regression model for reinfection the different effects of both age and sex observed in the two ethnic groups when exposure was not allowed for were no longer significant (ethnic group -age and ethnic group-sex interaction terms non-significant, *P* = 0. 06 and *P* = 0.10 respectively; Table 3). Furthermore, associations between age and reinfection strengthened and the convex relationship between age and reinfection became more apparent/prominent after adjusting for cercarial exposure (Table 3, *P = *0.02).

Table 4 shows the results of the logistic regression model adjusting for cercarial exposure, without an ethnic group-sex interaction, and with and without adjustment for plasma levels of IgE and IgG_4_ to the recombinant protein SmTAL1. Before controlling for SmTAL1 -IgE and -IgG_4_ levels but after controlling for cercarial exposure, age, but not ethnic group and sex, was significantly associated with reinfection. After controlling for levels of IgE and IgG_4_ to SmTAL1, however, age was also no longer significantly associated with reinfection (*P* = 0.09, Table 4). When age was removed from the logistic regression model (Table 5), SmTAL1-IgE levels were significantly negatively associated with reinfection at 12 months, whilst SmTAL1-IgG_4_ levels were significantly positively associated with reinfection. Similar patterns were observed, though not as strong, after controlling for levels of IgE and IgG_4_ to adult worm tegumental antigen (Teg) (results not shown). Age remained significantly associated with reinfection after adjusting for SWA -IgE and -IgG_4_, and SEA –IgE and –IgG_4_ (*P*≤0.03).

**Table 4:** Associations with reinfection before and after adjusting for IgE and IgG4 levels to TAL1.*

		Model 4		Model 5	
		OR** (CI_95%_)	*P*-value	OR** (CI_95%_)	*P*-value
**Ethnic group (Bagungu)**		0.74 (0.30, 1.79)	0.50	0.67 (0.22, 2.05)	0.49
**Sex (Male)**		0.82 (0.34, 2.00)	0.67	1.35 (0.45, 4.08)	0.59
**Age** a					
	7–9 years	1.00		1.00	
	10–12 years	0.53 (0.06, 4.43)		0.20 (0.02, 2.49)	
	13–16 years	0.57 (0.07, 4.58)		0.32 (0.02, 4.30)	
	17–23 years	0.33 (0.05, 2.17)		0.32 (0.03, 3.01)	
	24–30 years	0.12 (0.02, 0.77)		0.04 (0.003, 0.50)	
	31–38 years	0.16 (0.03, 1.00)		0.12 (0.01, 1.22)	
	39–50 years	0.07 (0.01, 0.49)	0.02	0.07 (0.01, 0.78)	0.09
**Cercarial exposure** † **^b^**		6.03 (3.24, 11.20)	<0.0001	7.18 (3.30, 15.65)	<0.0001
**SmTAL1-IgE** ‡ **^c^**				0.66 (0.27, 1.61)	0.35
**SmTAL1-IgG4** †† **^c^**				1.56 (0.64, 3.77)	0.31

*Associations were explored using multivariable logistic models for reinfection, controlling for ethnic group, sex, age, and cercarial exposure; individuals with detectable eggs at 5 weeks were excluded. Model 4 is before and Model 5 is after adjusting for levels of IgE and IgG4 to TAL1.

**OR  =  odds ratio.

a 6 degrees of freedom used to fit age.

**†:** Ln(exposure units +0.01); derived by weighting water contact duration by water contact site, time of day and degree of immersion.

b OR per unit S.D. increase.

**‡:** Ln (SmTAL1-IgE +0.03); antibody levels indicated by absorbance values (OD 490 nm).

c OR per unit increase.

**††:** Ln (SmTAL1-IgG4 +0.03); antibody levels indicated by absorbance values (OD 490 nm).

**Table 5:** Associations between IgE and IgG4 to TAL1 and *S. mansoni* reinfection*.

		OR** (CI_95%_)	*P*-value
**Ethnic group (Bagungu)**		0.72 (0.27, 1.90)	0.51
**Sex (Male)**		1.18 (0.44, 3.18)	0.74
**Cercarial exposure** † **^a^**		5.48 (2.85, 10.56)	<0.0001
**SmTAL1-IgE** ‡ **^b^**		0.42 (0.18, 0.96)	0.03
**SmTAL1-IgG4** †† **^b^**		2.28 (0.99, 5.22)	0.04

*Associations were explored using multivariable logistic models for reinfection, controlling for ethnic group, sex, and cercarial exposure; individuals with detectable eggs at 5 weeks were excluded.

**OR  =  odds ratio.

**†:** Ln(exposure units +0.01); derived by weighting water contact duration by water contact site, time of day and degree of immersion.

a OR per unit S.D. increase.

**‡:** Ln (SmTAL1-IgE +0.03); antibody levels indicated by absorbance values (OD 490 nm).

b OR per unit increase.

**††:** Ln (SmTAL1-IgG4 +0.03); antibody levels indicated by absorbance values (OD 490 nm).

## Discussion

Small-scale, micro-epidemiological studies investigating risk factors for *Schistosoma* infection are often limited by their lack of data on individual-level cercarial exposure (for example, [51]). Although some studies have attempted to overcome this limitation by using proxy markers for exposure such as distance to an infective water body [52], [53], care should be taken because exposure to *S. mansoni* infection is a highly complex, multi-factorial process [54] and unreliable exposure estimates may bias results [45]. In the current study, we used individual-measures of cercarial exposure derived from longitudinal observational and malacological studies to explore risk factors for infection in a Ugandan fishing community which had received treatment as part of a wider multi-disciplinary study investigating the effects of praziquantel treatment over time in a highly endemic community. We investigated whether there was any evidence for age, sex and ethnic background influencing susceptibility to reinfection in our study population and whether schistosome-specific IgE responses could account for any of the observed differences in reinfection. By attempting to capture the total ecology of our study population and taking a multivariable approach to adjust for individual variations in cercarial exposure, we were able to demonstrate that ethnicity- and sex- related variations in *S. mansoni* reinfection were likely to be due to variations in cercarial exposure, as opposed to biological differences in susceptibility to infection. Age-related differences in reinfection could not be explained by exposure, however and appeared linked/related to the balance between TAL1-IgE and TAL1-IgG_4_, which itself was significantly related to resistance to reinfection.

There are some limitations to consider when interpreting the results from this study. Firstly, although deemed the gold standard for measuring water contact behaviour [55], water contact observations are by no means perfect. Firstly, the presence of an observer may alter the behaviour of the study participants; the impact of this in the current study should have been minimised, however, since observations were conducted over an extended time period (11 months). A second limitation of direct observations is that they may be less accurate where water contacts are widely dispersed, for example, among fishing communities such as ours. Although there is some evidence that this may have been the case in Booma [18], [43], qualitative data collected subsequently suggest that missing fishing contacts are likely to have occurred away from the shoreline, in deeper waters that are not inhabited by *Biomphalaria* snails (Pinot de Moira, unpublished data) and therefore are unlikely to have been important in transmission. Issues such as these may be resolved with the advancement of global positioning system (GPS) technology [56].

The qualitative data collected subsequent to the water contact observations also suggest that behaviour in Booma may have altered since the observations were made: there is evidence that the Bagungu in particular have made a concerted effort to change their behaviour in order to avoid infection. Therefore, because water contact behaviour was only measured for the first 11 months following treatment, we decided to limit our analysis to reinfection observed during the first 12 months after treatment, so as not to bias results. Whilst limiting our analysis to the first 12 months of reinfection meant that we had to restrict analyses to a binary response (reinfected vs. not reinfected) due to the low levels of reinfection seen in our study population, it did have the advantage of being less subject to noise; immune responses measured 7 weeks after treatment are likely to be more closely related to reinfection at 12 months than at 18 and 22 months.

Reinfection was low in Booma, particularly given the high intensities and prevalence of infection seen before treatment. One explanation is a reduction in transmission as a result of treatment, as has been observed elsewhere [57]. Treatment may have also had a protective effect against heavy reinfection; in other schistosomiasis mansoni-endemic populations, substantially lower GM egg counts have been observed amongst reinfected individuals compared with newly infected individuals and Colley and Secor have proposed that multiple treatments and reinfections may induce immunological changes which lead to resistance to reinfection [58]. Alternatively, Kazibwe *et al.* noted that the level of the lake surrounding Booma is increasing, which seems to be influencing the population dynamics of the *Biomphalaria* species inhabiting the Lake and therefore possibly *S. mansoni* transmission [46]. Fluctuations in transmission due to natural environmental changes are frequently observed amongst schistosomiasis-endemic communities [59].

In the univariable analysis, sex was significantly associated with reinfection, with males having a greater likelihood of reinfection than females. Greater prevalence or intensities of infection amongst males have been documented elsewhere for all three of the major species of *Schistosoma* [19], [20], [22], [24], [60]. As stated previously, differences in schistosome infection amongst males and females are frequently attributed to gender-related differences in water contact behaviour [20], [23], [24]. There has been speculation, however, that sex may also influence susceptibility to infection [61], substantiated by studies in mice [25], [26] and also in humans, where gender-related differences in the magnitude of humoral immune responses to schistosomes have been reported [14], [27]. In Booma, the influence of sex on reinfection appeared to differ depending on ethnic background. Whereas the odds of reinfection were significantly greater amongst females in the Alur ethnic group, they were significantly lower amongst females in the Bagungu ethnic group. Similar sex-ethnicity interactions have been reported elsewhere [28]. Results from our analysis using individual estimates of cercarial exposure suggest that these differences in reinfection were due to variations in cercarial exposure, as opposed to differences in susceptibility to reinfection.

Similar to reports made by Kabatereine and colleagues [20] in the neighbouring village of Piida, prevalence of reinfection was significantly greater in the Alur, which, from multivariable analyses, appeared to be due to the relatively high levels of reinfection observed in the Alur females but the relatively low levels of reinfection observed amongst Bagungu females. Kabatereine and colleagues speculated that genetic-related differences in susceptibility to infection may have been the cause for their observed differences in infection intensity, which, given that the Alur make up approximately 1.6% of the Ugandan population (Uganda Bureau of Statistics, 2002 Uganda Population and Housing Census), could have important public health implications. From our analysis it would instead appear that the Alurs' apparent greater susceptibility to infection is due their greater exposure to infection. It is interesting to note, however, that variations in simple (unweighted) durations of water contact were not fully able to explain observed differences in reinfection: observed ethnic group-sex interactions were only removed once the risks associated with the time of day, the site of contact and degree of immersion during contact were incorporated into water contact durations. This is consistent with other studies [45], [62], and highlights the value of deriving exposure indices in order to more accurately reflect exposure.

Age-infection profiles in this study were typical of schistosomiasis-endemic communities: infection was greatest in children and comparably lower amongst adults. There has been some debate as to the relative importance of exposure vs. susceptibility to infection in explaining the characteristic age-infection curves observed in endemic communities [17], [63]. Kabatereine and colleagues observed post-adolescent declines in infection in Piida, despite adults remaining heavily exposed after childhood, suggesting that adults may have some kind of increased resistance to infection [18]. Unfortunately, however, Kabatereine and colleagues' study was an ecological study, where the unit of observation was the population as opposed to the individual. Here, using individual measures of cercarial exposure, we were able to corroborate Kabatereine and colleagues' findings and demonstrate that age remains significantly associated with reinfection even after allowing for individual variations in cercarial exposure; in fact, in our analysis, age-infection associations strengthened after adjusting for exposure. Thus, it appears that resistance to *Schistosoma* infection can develop; the fact that variations in exposure were able to explain sex- and ethnicity- related differences in reinfection in our study strengthens this conclusion.

Evidence from our study suggests that reductions in infection with age may be linked to changes in the balance of TAL1-IgE and TAL1-IgG_4_. This is in agreement with other studies that have indicated that whilst a strong IgE response to adult worm antigens may protect against schistosome infection, a strong IgG_4_ response directed against adult worm antigens may actually increase susceptibility to infection [10], [13], [38]. IgG_4_ is known to modulate the effects of IgE by attenuating the ability of IgE to mediate certain effector mechanisms, both in allergy[64] and parasitic infections [65]. We were unable, however, to disentangle the confounding between the TAL1-IgE and TAL1-IgG_4_ balance and age; therefore it is not clear whether changes in the balance of these antibodies with age are due to prolonged exposure to antigen, possibly following worm death[66], or age-related changes in the immune system. In support of the former hypothesis, whilst antibody responses to TAL1, an antigen predominately expressed in the adult worm, tend to increase with age, antibody responses to the egg antigen TAL2 (formerly Sm21.7), which is continuously released during infection, are unchanged with age [67]. Evidence also comes from the so-called “peak shift”, where infection intensity peaks at a younger age in higher transmission areas [68]–[70]. A similar phenomenon was observed here, whereby infection was greater and peaked earlier in the more heavily exposed Alur children than in the less-exposed Bagungu children. However, age-infection profiles typical of schistosomiasis-endemic populations have also been observed to develop among recently exposed, immunological naive populations [71], suggesting that there is also an age-dependent component of acquired immunity to *Schistosoma* spp. infections.

In summary, this study took a multidisciplinary approach, drawing on malacological, ecological and behavioural data in order to capture the ecology of our study population. Using previously constructed individual-level exposure scores and multivariable methods, we were able to remove noise introduced due to variations in exposure and thus able to identify potentially immune individuals. We found that exposure to infection varied dramatically depending on both ethnic background and sex, which created strong ethnic- and gender-related differences in infection. While sex is routinely considered an *a priori* confounder in immuno-epidemiological studies, ethnic background is rarely considered, which could have strong implications for study outcomes; we therefore recommend that ethnic background is included as an *a priori* confounder in all immuno-epidemiological studies. Our study also corroborates previous evidence that the reductions in schistosome infection intensity observed amongst adults in endemic communities are due to acquired immunity to infection; evidence suggests that this could be linked to changes in the balance of IgE and IgG_4_ to tegumental antigens. These findings highlight the benefit of employing multidisciplinary methodologies to capture and adjust for patterns of exposure in population-based studies where confounders are a major limitation; this will enable firmer conclusions to be drawn regarding the biology of disease, even in complex settings such as ours.

## Supporting Information

Checklist S1 STROBE Checklist
(0.03 MB XLS)

Tables S1 Comparison tables of the analysis with and without 5 week egg-positive individuals.
(0.18 MB DOC)
