# Supplementary material for: Analysis of Complex Patterns of Human Exposure and Immunity to Schistosomiasis mansoni: The Influence of Age, Sex, Ethnicity and IgE
Source: PLoS Negl Trop Dis. 2010 Sep 14;4(9):e820. doi: 10.1371/journal.pntd.0000820 (PMC2939029; doi:10.1371/journal.pntd.0000820)
Supplement: Tables S1 — Comparison tables of the analysis with and without 5 week egg-positive individuals. (0.18 MB DOC) [file pntd.0000820.s002.doc]

**Supporting Tables S1 - Comparison tables of the analysis with and without 5 week egg-positive individuals**

## Table 1. Associations between potential risk factors and *S. mansoni* reinfection 12 months after treatment

## A. All individuals, including those with detectable eggs at 5 weeks

|  |  | **N (%) or GM (GSD)*** | **N (%) reinfected** | **OR** (CI_95%_)** | ***P*-value** |
| --- | --- | --- | --- | --- | --- |
| **Ethnic group** | Alur | 87 (40.7) | 54 (62.1) | 1.00 |  |
|  | Bagungu | 127 (59.4) | 55 (43.3) | 0.47 (0.27, 0.82) | 0.01 |
| **Sex** | Female | 96 (44.9) | 38 (39.6) | 1.00 |  |
|  | Male | 118 (55.1) | 71 (60.2) | 2.31 (1.33, 4.00) | 0.03 |
| **Age (years)** | 7-9 | 26 (12.2) | 17 (65.4) | 1.00 |  |
|  | 10-12 | 33 (15.4) | 27 (81.8) | 2.38 (0.72, 7.89) |  |
|  | 13-16 | 24 (11.2) | 18 (75.0) | 1.59 (0.47, 5.42) |  |
|  | 17-23 | 24 (11.2) | 10 (41.7) | 0.38 (0.12, 1.19) |  |
|  | 24-30 | 34 (15.9) | 12 (35.3) | 0.29 (0.10, 0.84) |  |
|  | 31-38 | 37 (17.3) | 14 (37.8) | 0.32 (0.11, 0.92) |  |
|  | 39-50 | 36 (16.8) | 11 (30.6) | 0.23 (0.08, 0.68) | <0.0001 |
| **Water contact duration^†a^** | | 83.09 (6.24) | - | 2.92 (1.99, 4.28) | <0.0001 |
| **Cercarial exposure^‡a^** | | 1.10 (24.50) | - | 3.07 (2.17, 4.35) | <0.0001 |

## B. Excluding individuals with detectable eggs at 5 weeks

|  |  | **N (%) or GM (GSD)*** | **N (%) reinfected** | **OR** (CI_95%_)** | ***P*-value** |
| --- | --- | --- | --- | --- | --- |
| **Ethnic group** | Alur | 57 (35.0) | 29 (50.9) | 1.00 |  |
|  | Bagungu | 106 (65.0) | 38 (35.9) | 0.54 (0.28, 1.04) | 0.06 |
| **Sex** | Female | 74 (45.4) | 23 (31.1) | 1.00 |  |
|  | Male | 89 (54.6) | 44 (49.4) | 2.17 (1.14, 4.13) | 0.02 |
| **Age (years)** | 7-9 | 15 (9.2) | 7 (46.7) | 1.00 |  |
|  | 10-12 | 15 (9.2) | 9 (60.0) | 1.71 (0.40, 7.29) |  |
|  | 13-16 | 16 (9.8) | 11 (68.8) | 2.51 (0.58, 10.88) |  |
|  | 17-23 | 23 (14.1) | 10 (43.5) | 0.88 (0.24, 3.25) |  |
|  | 24-30 | 30 (18.4) | 10 (33.3) | 0.57 (0.16, 2.03) |  |
|  | 31-38 | 35 (21.5) | 14 (40.0) | 0.76 (0.23, 2.58) |  |
|  | 39-50 | 29 (17.8) | 6 (20.7) | 0.30 (0.08, 1.16) |  |
| **Water contact duration^†a^** | | 84.34 (6.4) |  | 4.19 (2.50, 7.04) | <0.0001 |
| **Cercarial exposure^‡a^** | | 1.08 (25.9) |  | 4.80 (2.89, 7.95) | <0.0001 |

*GM = geometric mean, GSD = geometric standard deviation

**OR = odds ratio

^†^ Ln (minutes + 1)

^a^ ORs are per unit S.D. increase

^‡^ Ln(exposure units + 0.01); derived by weighting water contact duration by the average number of infected snails at water contact site, time of day and degree of immersion

## Table 2. Associations between post-treatment IgE and IgG4 against worm and egg, and 12 month reinfection

## A. All individuals, including those with detectable eggs at 5 weeks

|  |  | **OR* (CI_95%_)** | ***P*-value** |
| --- | --- | --- | --- |
| **IgE **** | |  |  |
|  | SWA | 0.89 (0.61, 1.30) | 0.55 |
|  | Tegument | 0.84 (0.56, 1.26) | 0.40 |
|  | SmTAL1 | 0.92 (0.69, 1.21) | 0.54 |
|  | SEA | 1.28 (0.60, 2.74) | 0.52 |
| **IgG4**** | |  |  |
|  | SWA | 1.06 (0.76, 1.49) | 0.72 |
|  | Tegument | 1.08 (0.68, 1.73) | 0.73 |
|  | SmTAL1 | 1.05 (0.80, 1.37) | 0.74 |
|  | SEA | 1.32 (0.72, 2.39) | 0.36 |

## B. Excluding individuals with detectable eggs at 5 weeks

|  |  | **OR* (CI_95%_)** | ***P*-value** |
| --- | --- | --- | --- |
| **IgE **** | |  |  |
|  | SWA | 1.14 (0.73, 1.77) | 0.57 |
|  | Tegument | 1.16 (0.71, 1.91) | 0.55 |
|  | SmTAL1 | 0.96 (0.69, 1.33) | 0.81 |
|  | SEA | 1.75 (0.71, 4.36) | 0.23 |
| **IgG4**** | |  |  |
|  | SWA | 1.17 (0.78, 1.76) | 0.45 |
|  | Tegument | 1.11 (0.64, 1.94) | 0.71 |
|  | SmTAL1 | 1.10 (0.80, 1.51) | 0.55 |
|  | SEA | 1.07 (0.54, 2.11) | 0.85 |

*OR = odds ratio

**OR per unit increase; antibody levels were estimated by isotype-specific ELISA, OD values were log-transformed with 0.03 added to remove zeros.

## Table 3. Results from multivariable logistic models examining associations between demographic factors and 12 month reinfection*

## A. All individuals, including those with detectable eggs at 5 weeks

|  |  | **Model 1^a^** | | **Model 2^b^** | | **Model 3^c^** | |
| --- | --- | --- | --- | --- | --- | --- | --- |
|  |  | **OR** (CI_95%_)** | ***P*-value** | **OR** (CI_95%_)** | ***P*-value** | **OR** (CI_95%_)** | ***P*-value** |
| **Ethnic group (Bagungu)** | | 0.08 (0.02, 0.26) | <0.0001 | 0.14 (0.04, 0.48) | 0.001 | 0.30 (0.08, 1.13) | 0.07 |
| **Sex (Male)** | | 0.30 (0.09, 0.93) | 0.03 | 0.59 (0.16, 2.12) | 0.42 | 0.35 (0.10, 1.22) | 0.09 |
| **Ethnicity x sex interaction** | | 27.49 (5.98, 126.26) | <0.0001 | 7.22 (1.28, 40.82) | 0.02 | 4.98 (0.86, 28.92) | 0.07 |
| **Age^d^** | |  |  |  |  |  |  |
|  | 7-9 years | 1.00 |  | 1.00 |  | 1.00 |  |
|  | 10-12 years | 1.36 (0.28, 6.66) |  | 1.62 (0.28, 9.47) |  | 0.71 (0.09, 5.47) |  |
|  | 13-16 years | 1.42 (0.28, 7.19) |  | 1.41 (0.24, 8.25) |  | 0.59 (0.08, 4.35) |  |
|  | 17-23 years | 0.71 (0.16, 3.15) |  | 0.94 (0.18, 4.97) |  | 0.37 (0.06, 2.39) |  |
|  | 24-30 years | 0.38 (0.09, 1.61) |  | 0.31 (0.06, 1.58) |  | 0.13 (0.02, 0.85) |  |
|  | 31-38 years | 0.42 (0.11, 1.68) |  | 0.30 (0.06, 1.44) |  | 0.15 (0.02, 0.93) |  |
|  | 39-50 years | 0.23 (0.05, 1.00) | 0.05 | 0.18 (0.03, 0.96) | 0.02 | 0.07 (0.01, 0.48) | 0.01 |
| **Water contact duration^†e^** | |  |  | 4.11 (2.33, 7.24) | <0.0001 |  |  |
| **Cercarial exposure^‡e^** | |  |  |  |  | 5.03 (2.74, 9.25) | <0.0001 |

*Models examined associations between ethnicity, sex and age, and *S. mansoni* reinfection 12 months after treatment. Model 1 is without adjustment for behaviour or cercarial exposure, Model 2 adjusts for observed water contact duration and Model 3 adjusts for estimated cercarial exposure.

^a^ Model included ethnic group, sex, age and infection intensity at 5 weeks [ln(epg + 1), used to as an indicator of treatment efficacy]

^b^ Model included ethnic group, sex, age, infection intensity at 5 weeks [ln(epg + 1), used to as an indicator of treatment efficacy] and observed water contact duration

^c^ Model included ethnic group, sex, age, infection intensity at 5 weeks [ln(epg + 1), used to as an indicator of treatment efficacy] and cercarial exposure score

**OR = odds ratio

^d^ 6 degrees of freedom used to fit age

^†^ Ln (minutes + 1)

^e^ OR per unit S.D. increase

^‡^ Ln(exposure units + 0.01); derived by weighting water contact duration by water contact site, time of day and degree of immersion

## B. Excluding individuals with detectable eggs at 5 weeks

|  | **Model 1^a^** | | **Model 2^b^** | | **Model 3^c^** | |
| --- | --- | --- | --- | --- | --- | --- |
|  | **OR* (CI_95%_)** | ***P*-value** | **OR* (CI_95%_)** | ***P*-value** | **OR* (CI_95%_)** | ***P*-value** |
| **Ethnic group (Bagungu)** | 0.08 (0.02, 0.27) | <0.0001 | 0.14 (0.04, 0.53) | 0.002 | 0.31 (0.08, 1.23) | 0.09 |
| **Sex (Male)** | 0.32 (0.10, 1.03) | 0.05 | 0.66 (0.18, 2.46) | 0.54 | 0.39 (0.11, 1.39) | 0.14 |
| **Ethnicity x sex interaction** | 27.18 (5.67, 130.35) | <0.0001 | 6.80 (1.13, 40.88) | 0.04 | 4.65 (0.75, 28.83) | 0.10 |
| **Age^d^** |  |  |  |  |  |  |
| 7-9 years | 1.00 |  | 1.00 |  | 1.00 |  |
| 10-12 years | 1.17 (0.23, 6.08) |  | 1.40 (0.23, 8.49) |  | 0.51 (0.06, 4.37) |  |
| 13-16 years | 1.85 (0.34, 10.02) |  | 1.63 (0.27, 9.98) |  | 0.67 (0.08, 5.35) |  |
| 17-23 years | 0.72 (0.16, 3.26) |  | 0.95 (0.18, 5.03) |  | 0.37 (0.06, 2.42) |  |
| 24-30 years | 0.38 (0.09, 1.64) |  | 0.32 (0.06, 1.62) |  | 0.13 (0.02, 0.85) |  |
| 31-38 years | 0.43 (0.10, 1.73) |  | 0.31 (0.06, 1.50) |  | 0.15 (0.02, 0.93) |  |
| 39-50 years | 0.22 (0.05, 1.03) | 0.05 | 0.19 (0.03, 1.03) | 0.03 | 0.07 (0.01, 0.51) | 0.02 |
| **Water contact duration^†e^** |  |  | 3.87 (2.18, 6.90) | <0.0001 |  |  |
| **Cercarial exposure^‡e^** |  |  |  |  | 5.12 (2.71, 9.66) | <0.0001 |

*Models examined associations between ethnicity, sex and age, and *S. mansoni* reinfection 12 months after treatment. Model 1 is without adjustment for behaviour or cercarial exposure, Model 2 adjusts for observed water contact duration and Model 3 adjusts for estimated cercarial exposure.

^a^ Model included ethnic group, sex and age; excludes individuals with a detectable egg count at 5 weeks

^b^ Model included ethnic group, sex, age and observed water contact duration; excludes individuals with a detectable egg count at 5 weeks

^c^ Model included ethnic group, sex, age and cercarial exposure score; excludes individuals with a detectable egg count at 5 weeks

**OR = odds ratio

^d^ 6 degrees of freedom used to fit age

^†^ Ln (minutes + 1)

^e^ OR per unit S.D. increase

^‡^ Ln(exposure units + 0.01); derived by weighting water contact duration by water contact site, time of day and degree of immersion

## Table 4. Associations with reinfection before and after adjusting for IgE and IgG4 levels to TAL1*

## A. All individuals, including those with detectable eggs at 5 weeks

|  |  | **Model 4** |  | **Model 5** |  |
| --- | --- | --- | --- | --- | --- |
|  |  | **OR** (CI_95%_)** | ***P*-value** | **OR* (CI_95%_)** | ***P*-value** |
| **Ethnic group (Bagungu)** | | 0.73 (0.30, 1.75) | 0.48 | 0.66 (0.22, 1.94) | 0.44 |
| **Sex (Male)** | | 0.78 (0.33, 1.84) | 0.58 | 1.25 (0.45, 3.52) | 0.67 |
| **Age^a^** | |  |  |  |  |
|  | 7-9 years | 1.00 |  | 1.00 |  |
|  | 10-12 years | 0.73 (0.10, 5.53) |  | 0.37 (0.04, 3.68) |  |
|  | 13-16 years | 0.51 (0.07, 3.79) |  | 0.33 (0.03, 3.75) |  |
|  | 17-23 years | 0.34 (0.05, 2.14) |  | 0.38 (0.04, 3.31) |  |
|  | 24-30 years | 0.12 (0.02, 0.77) |  | 0.05 (0.004, 0.59) |  |
|  | 31-38 years | 0.17 (0.03, 1.00) |  | 0.16 (0.02, 1.46) |  |
|  | 39-50 years | 0.07 (0.01, 0.46) | 0.01 | 0.08 (0.01, 0.78) | 0.08 |
| **Cercarial exposure^†b^** | | 5.90 (3.26, 10.68) | <0.0001 | 6.57 (3.23, 13.36) | <0.0001 |
| **SmTAL1-IgE^‡c^** | |  |  | 0.58 (0.25, 1.34) | 0.19 |
| **SmTAL1-IgG4^††c^** | |  |  | 1.69 (0.74, 3.86) | 0.20 |

*Associations were explored using multivariable logistic models for reinfection, controlling for ethnicity, sex, age, treatment efficacy [ln(epg + 1) at 5 weeks] and cercarial exposure; Model 4 is before and Model 5 is after adjusting for levels of IgE and IgG4 to TAL1

**OR = odds ratio

^a^ 6 degrees of freedom used to fit age

^†^ Ln(exposure units + 0.01); derived by weighting water contact duration by water contact site, time of day and degree of immersion

^b^OR per unit S.D. increase

**^‡^** Ln (SmTAL1-IgE + 0.03); antibody levels indicated by absorbance values (OD 490nm)

^c^ OR per unit increase

## ^††^ Ln (SmTAL1-IgG4 + 0.03); antibody levels indicated by absorbance values (OD 490nm)

## B. Excluding individuals with detectable eggs at 5 weeks

|  |  | **Model 4** |  | **Model 5** |  |
| --- | --- | --- | --- | --- | --- |
|  |  | **OR* (CI_95%_)** | ***P*-value** | **OR* (CI_95%_)** | ***P*-value** |
| **Ethnic group (Bagungu)** | | 0.74 (0.30, 1.79) | 0.50 | 0.67 (0.22, 2.05) | 0.49 |
| **Sex (Male)** | | 0.82 (0.34, 2.00) | 0.67 | 1.35 (0.45, 4.08) | 0.59 |
| **Age^a^** | |  |  |  |  |
|  | 7-9 years | 1.00 |  | 1.00 |  |
|  | 10-12 years | 0.53 (0.06, 4.43) |  | 0.20 (0.02, 2.49) |  |
|  | 13-16 years | 0.57 (0.07, 4.58) |  | 0.32 (0.02, 4.30) |  |
|  | 17-23 years | 0.33 (0.05, 2.17) |  | 0.32 (0.03, 3.01) |  |
|  | 24-30 years | 0.12 (0.02, 0.77) |  | 0.04 (0.003, 0.50) |  |
|  | 31-38 years | 0.16 (0.03, 1.00) |  | 0.12 (0.01, 1.22) |  |
|  | 39-50 years | 0.07 (0.01, 0.49) | 0.02 | 0.07 (0.01, 0.78) | 0.09 |
| **Cercarial exposure**^†^**^b^** | | 6.03 (3.24, 11.20) | <0.0001 | 7.18 (3.30, 15.65) | <0.0001 |
| **SmTAL1-IgE^‡c^** | |  |  | 0.66 (0.27, 1.61) | 0.35 |
| **SmTAL1-IgG4^††c^** | |  |  | 1.56 (0.64, 3.77) | 0.31 |

*Associations were explored using multivariable logistic models for reinfection, controlling for ethnicity, sex, age, and cercarial exposure; individuals with a detectable egg count at 5 weeks were excluded. Model 4 is before and Model 5 is after adjusting for levels of IgE and IgG4 to TAL1

**OR = odds ratio

^a^ 6 degrees of freedom used to fit age

^†^ Ln(exposure units + 0.01); derived by weighting water contact duration by water contact site, time of day and degree of immersion

^b^OR per unit S.D. increase

**^‡^** Ln (SmTAL1-IgE + 0.03); antibody levels indicated by absorbance values (OD 490nm)

^c^ OR per unit increase

## ^††^ Ln (SmTAL1-IgG4 + 0.03); antibody levels indicated by absorbance values (OD 490nm)

## Table 5. Associations between IgE and IgG4 to TAL1 and *S. mansoni* reinfection*

## A. All individuals, including those with detectable eggs at 5 weeks

|  |  | **OR** (CI_95%_)** | ***P*-value** |
| --- | --- | --- | --- |
| **Ethnic group (Bagungu)** | | 0.68 (0.27, 1.72) | 0.42 |
| **Sex (Male)** | | 1.26 (0.49, 3.21) | 0.63 |
| **Cercarial exposure^†a^** | | 5.06 (2.76, 9.27) | <0.0001 |
| **SmTAL1-IgE^‡b^** | | 0.39 (0.18, 0.86) | 0.02 |
| **SmTAL1-IgG4^††b^** | | 2.29 (1.04, 5.03) | 0.03 |

* Associations were explored using multivariable logistic models for reinfection, controlling for ethnicity, sex, treatment efficacy [ln(epg + 1) at 5 weeks] and cercarial exposure

**OR = odds ratio

^†^ Ln(exposure units + 0.01); derived by weighting water contact duration by water contact site, time of day and degree of immersion

^a^ OR per unit S.D. increase

^‡^ Ln (SmTAL1-IgE + 0.03); antibody levels indicated by absorbance values (OD 490nm)

^b^ OR per unit increase

## ^††^ Ln (SmTAL1-IgG4 + 0.03); antibody levels indicated by absorbance values (OD 490nm)

## B. Excluding individuals with detectable eggs at 5 weeks

|  |  | **OR** (CI_95%_)** | ***P*-value** |
| --- | --- | --- | --- |
| **Ethnic group (Bagungu)** | | 0.72 (0.27, 1.90) | 0.51 |
| **Sex (Male)** | | 1.18 (0.44, 3.18) | 0.74 |
| **Cercarial exposure**^†^**^a^** | | 5.48 (2.85, 10.56) | <0.0001 |
| **SmTAL1-IgE^‡b^** | | 0.42 (0.18, 0.96) | 0.03 |
| **SmTAL1-IgG4^††b^** | | 2.28 (0.99, 5.22) | 0.04 |

* Associations were explored using multivariable logistic models for reinfection, controlling for ethnicity, sex, and cercarial exposure; individuals with a detectable egg count at 5 weeks were excluded.

**OR = odds ratio

^†^ Ln(exposure units + 0.01); derived by weighting water contact duration by water contact site, time of day and degree of immersion

^a^ OR per unit S.D. increase

^‡^ Ln (SmTAL1-IgE + 0.03); antibody levels indicated by absorbance values (OD 490nm)

^b^ OR per unit increase

## ^††^ Ln (SmTAL1-IgG4 + 0.03); antibody levels indicated by absorbance values (OD 490nm)
